# Innate myeloid cell TNFR1 mediates first line defence against primary *Mycobacterium tuberculosis* infection.

**DOI:** 10.1038/srep22454

**Published:** 2016-03-02

**Authors:** Noria Segueni, Sulayman Benmerzoug, Stéphanie Rose, Amandine Gauthier, Marie-Laure Bourigault, Flora Reverchon, Amandine Philippeau, François Erard, Marc Le Bert, Hélène Bouscayrol, Thierry Wachter, Irène Garcia, George Kollias, Muazzam Jacobs, Bernhard Ryffel, Valerie F.J. Quesniaux

**Affiliations:** 1 CNRS, UMR7355, Experimental and Molecular Immunology and Neurogenetics, 45071 Orleans, France; 2 University of Orleans, 45000 Orleans, France; 3 Oncology–radiotherapy Department, Regional Hospital, Orleans, France; 4 Department of Pathology and Immunology, University of Geneva Medical School, Geneva, Switzerland; 5 Institute for Immunology, Biomedical Sciences Research Center “Alexander Fleming”, Vari, Greece; 6 Division of Immunology, Department of Pathology and Institute of Infectious Disease and Molecular Medicine, Health Sciences Faculty, University of Cape Town, South Africa; 7 National Health Laboratory Service, South Africa

## Abstract

TNF is crucial for controlling *Mycobacterium tuberculosis* infection and understanding how will help immunomodulating the host response. Here we assessed the contribution of TNFR1 pathway from innate myeloid versus T cells. We first established the prominent role of TNFR1 in haematopoietic cells for controlling *M. tuberculosis* in TNFR1 KO chimera mice. Further, absence of TNFR1 specifically on myeloid cells (M-TNFR1 KO) recapitulated the uncontrolled *M. tuberculosis* infection seen in fully TNFR1 deficient mice, with increased bacterial burden, exacerbated lung inflammation, and rapid death. Pulmonary IL-12p40 over-expression was attributed to a prominent CD11b^+^ Gr1^high^ cell population in infected M-TNFR1 KO mice. By contrast, absence of TNFR1 on T-cells did not compromise the control of *M. tuberculosis* infection over 6-months. Thus, the protective TNF/TNFR1 pathway essential for controlling primary *M. tuberculosis* infection depends on innate macrophage and neutrophil myeloid cells, while TNFR1 pathway in T cells is dispensable.

Tumor necrosis factor alpha (TNF) is a major player in the host response to *Mycobacterium tuberculosis*. Tuberculosis (TB) is still a major health problem, reemerging in developed countries, with new resistant strains increasing the threat^1^. From one-third of the global population considered to be infected, 5% to 10% will develop an active disease, indicating that a robust host immune system can efficiently control the infection, although mycobacteria remain present for years in a latent form^2^,^3^. Immunomodulating the endogenous host defences may represent an interesting avenue to increase the arsenal against increasingly drug resistant *M. tuberculosis* infection. Coordinated innate and adaptive immune responses including T cells, macrophages, and the expression of mediators such as IFNγ, TNF, IL-1, IL-12p40, nitric oxide, reactive oxygen and nitrogen intermediates, are required to efficiently control *M. tuberculosis* infection^4^,^5^,^6^,^7^,^8^,^9^.

Immunodepression of the host such as CD4 T cell depletion during HIV co-infection can favour TB reactivation, and neutralization of TNF for the treatment of severe inflammatory diseases has been associated with reactivation of latent TB and increased susceptibility to primary TB infection^10^,^11^,^12^,^13^,^14^. Although poor health status and immune defences are recognized risk factors, the relative contribution of host innate versus adaptive immune responses for protection against primary tuberculosis infection remains poorly defined. The pivotal role of TNF, which is expressed and signals in both innate and adaptive immune cells, in these responses, deserves further attention.

TNF derived from hematopoietic cells rather than from stromal origin is essential for a normal host response to *M. bovis* BCG^15^ and we showed recently that myeloid and T-cells are the primary sources of TNF for host control of *M. tuberculosis*, TNF from myeloid cells being implicated in the early immune response while T-cell derived TNF was essential to sustain protection during chronic infection^16^.

TNF homotrimers signal through TNF receptors TNFRp55 (TNFR1) and TNFRp75 (TNFR2), membrane bound TNF preferentially signalling through TNFR2, and soluble TNF through TNFR1^17^,^18^. In addition, homotrimeric lymphotoxin α (LTα) also signals through TNFR1 and TNFR2. LTα was found to be dispensable for host control of acute *M. tuberculosis* infection using “neo-free” LTα^−/−^ mice with unperturbed TNF expression, although LTα might contribute to control chronic infection^19^. Membrane expressed TNF allowed cell-cell signalling and control of acute *M. tuberculosis* infection although long-term infection control additionally required soluble TNF^20^. The partial protection conferred by membrane TNF was attributed to signalling through TNFR2^21^.

TNFR1 was long recognized as essential for mounting the host response to *M. tuberculosis*^22^, while TNFR2 down-modulates protective immune function, through shedding and neutralisation of bioactive TNF^23^. TNFR1 expression is ubiquitous, allowing for some autocrine effects on macrophages or T cells for instance, or paradoxical anti-inflammatory effects on IL-12p40 expression in macrophages and dendritic cells^24^. Here, we addressed the relative contribution of TNFR1 mediated response in innate myeloid cells, versus adaptive T lymphocytes, or parenchymal, non-hematopoietic radioresistant cells, for the host control of *M. tuberculosis* infection. We show the prominent role of TNF/TNFR1 pathway in innate macrophage and neutrophil myeloid cells for controlling primary *M. tuberculosis* infection while TNFR1 pathway in T cells is dispensable.

## Results

### TNFR1 expressed on hematopoietic cells confers resistance to *M. tuberculosis* infection

**Figure 1:**
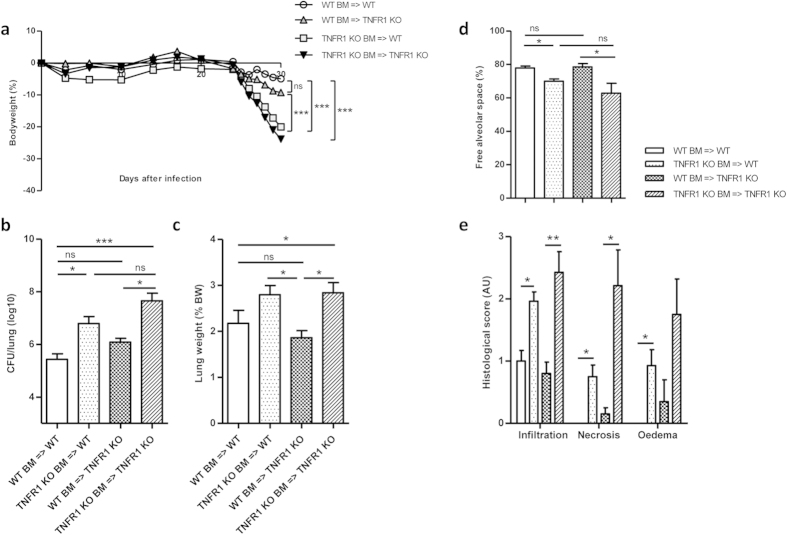
TNFR1 pathway on haematopoietic cells is crucial to control *M.tuberculosis* infection. TNFR1 deficient mice were lethally irradiated and reconstituted with bone marrow from either Ly5.1 WT mice (WT BM = > TNFR1 KO) or TNFR1 KO mice (TNFR1 KO BM = > TNFR1 KO) before *M.tuberculosis* intranasal infection. As controls, Ly5.2 C57Bl6 mice were irradiated and reconstituted with Ly5.1 WT BM (WT BM = > WT), or irradiated Ly5.1WT received TNFR1 KO BM (TNFR1 KO BM = > WT). Experimental groups were monitored for bodyweight (**a**). Lung bacterial load (**b**) and inflammation (**c**) were determined 30 days after infection. Lungs were harvested and fixed in 4% formol for HE staining. Bar graphs (**d, e**) summarize free alveolar space and scores of cell infiltration, necrosis and oedema. Results are expressed as mean +/− SEM (n = 6–7 mice per group). p < 0,05* p < 0,01** p < 0,001***.

TNFR1 deficient mice are extremely sensitive to virulent *M. tuberculosis* H37Rv infection^22^,^23^, similar to TNF deficient mice^25^,^26^. To assess the relative contribution of TNFR1 from hematopoietic versus stromal cell origins for the host control of *M. tuberculosis* infection, we first created chimeric mice deficient for TNFR1 on different cell compartments. TNFR1 deficient and WT mice were lethally irradiated and reconstituted with bone-marrow cells (BM) from either TNFR1 KO or WT mice. After 8 weeks of hematopoietic reconstitution mice were infected with *M. tuberculosis* H37Rv (1000 ± 200 CFU, i.n.). TNFR1 KO mice reconstituted with TNFR1 KO BM cells (TNFR1 KO BM = > TNFR1 KO) were extremely susceptible to infection, they lost weight rapidly and had to be terminated at day 30 post-infection (Fig. 1a). Interestingly, TNFR1 KO BM cells transferred the sensitive phenotype to WT mice (TNFR1 KO BM = > WT). Conversely, the sensitive phenotype of TNFR1 deficient mice was significantly corrected after reconstitution with WT BM (WT BM = > TNFR1 KO). Indeed, lung bacterial load and lung weight as indicator of pulmonary inflammation were significantly increased in mice reconstituted with TNFR1 KO BM, as compared to WT BM, irrespective of the genotype of the recipient (Fig. 1b,c), while transfer of WT BM to TNFR1 KO mice restored the phenotype with no significant difference as compared to WT BM = > WT control mice on day 30 post-infection. Free alveolar space, lung cell infiltration, necrosis and oedema were assessed histologically. Absence of TNFR1 on hematopoietic cells resulted in reduced alveolar space associated with an increased infiltration of inflammatory cells in the lungs, large necrotic areas within granulomatous structures and oedema in the lung tissue (Fig. 1d,e). On the contrary, TNFR1 KO mice reconstituted with WT BM showed no significant difference in lung pathophysiology as compared to WT BM = > WT controls at this time point. Thus, TNFR1 expressed on hematopoietic cells, and not on radio-resistant, parenchymal cells is central for the control of acute *M. tuberculosis* infection.

### Response to mycobacteria in myeloid cells deficient for TNFRI expression

We next assessed the relative contribution of the different hematopoietic cell populations to the TNFR1 mediated immune response to *M. tuberculosis* infection. We intercrossed Tnfrsf1α conditional knockout mice (*Tnfrsf1*α^*fl/fl*^) generated as described^27^ to LysM-Cre mice, leading to p55 TNFR1 inactivation in myeloid cells (*Tnfrsf1*α^*fl/fl*^ *LysM*^*cre/wt*^, M-TNFR1 KO). The lack of TNFR1 expression in these mice was verified by flow cytometry on bone marrow-derived macrophages or *ex vivo* in splenic granulocytic CD11b^+^ Ly6G^+^ cells and monocytic CD11b^+^ F4/80^+^ cells, as compared to CD8^+^ T lymphocytes, from systemically infected M-TNFR1 deficient mice highly sensitive to *M. bovis* BCG infection (See Supplementary Fig. S1A–D). The absence of TNFR1 did not compromise the macrophages inflammatory response to *M. tuberculosis* H37Rv or *M. bovis* BCG *in vitro* in terms of IL-12p40, TNF or CXCL1/KC release (Supplementary Fig. S1E). As a functional assay for TNFR1 activity we next assessed NO release in response to TNF. NO release in response to LPS was strongly reduced in the absence of TNFR1 in macrophages from M-TNFR1 KO or complete TNFR1 KO mice, or in the absence of TNF itself in TNF KO macrophages, which could be restored by supplementation with TNF in the latter cultures (Supplementary Fig. S1F).

Thus, the absence of TNFR1 signalling did not compromise macrophage inflammatory responses to mycobacteria in terms of IL-12 p40, TNF, or CXCL1 release, although it impaired NO release, a known mycobactericidal effector.

### Lethal *M. tuberculosis* infection in the absence of TNFR1 on myeloid cells

**Figure 2:**
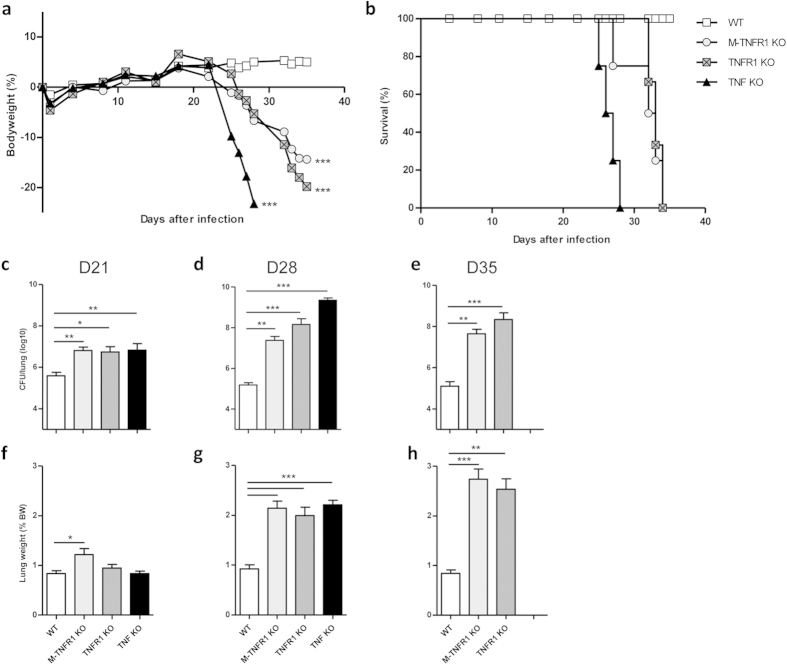
M-TNFR1 KO mice are unable to control acute *M. tuberculosis* infection. Mice deficient for TNFα, TNFR1 or specifically deficient for TNFR1 in myeloid cells (M-TNFR1 KO) and wild-type mice were exposed to an aerogenic dose of *M. tuberculosis* H37Rv (1000 CFU/mouse i.n.) and monitored for relative body weight gain (**a**) and survival (**b**). Data are means from n = 15–24 mice per group pooled from 4 independent experiments. Pulmonary bacterial load (**c–e**) and lung wet weight (**f–h**) were quantified after 21 days (**c,f**), 28 days (**d,g**) and 35 days (**e,h**) of infection. Results are expressed as mean +/− SEM (n = 7–10 from 2 experiments at day 21, n = 13–24 from 4 experiments at day 28 and n = 8–16 from 3 experiments at day 35). *p < 0.05; **p < 0.01; ***p < 0.001.

To determine the role of myeloid cell TNFR1 pathway on host control of tuberculosis *in vivo*, we next infected TNF-, TNFR1- or M-TNFR1 KO mice with *M. tuberculosis* and compared outcomes with WT control mice. Mice deficient for TNFR1 on myeloid cells rapidly lost weight and succumbed by day 35 after infection with *M. tuberculosis* H37Rv, similar to fully deficient TNFR1 KO mice, while wild-type controls survived the acute phase of infection, and fully TNF deficient mice succumbed even earlier by day 28 (Fig. 2a,b). Impairment in TNF or the TNFR1 pathway led to increased bacterial loads in these animals. Mice fully deficient for TNFR1 or myeloid cell specific M-TNFR1 KO mice exhibited significant increase in pulmonary bacterial loads already on day 21, similar to TNF deficient mice (Fig. 2c), with high bacterial burdens (over 2 log10 increase) on day 28 or 35 (Fig. 2d,e). Although complete absence of TNF seemed most drastic on day 28, shortly before the mice succumbed, absence of TNFR1 restricted to myeloid cells in M-TNFR1 KO mice yielded a similar defective bacterial control as the complete absence of TNFR1. TNF and TNFR1 deficient mice exhibited strongly increased lung weights, an indicator of lung inflammation, 4 to 5 weeks post-infection (Fig. 2f–h). Interestingly, TNFR1 deficiency restricted to myeloid cells in M-TNFR1 KO mice recapitulated this increased lung inflammation which was already detectable at 3 weeks post-infection (Fig. 2f).

Therefore, mice deficient for TNFR1 on myeloid cells are unable to control *M. tuberculosis* infection and develop a rapid, exacerbated lung inflammation, similar to complete TNFR1 deficient mice.

### Absence of TNFR1 on myeloid cells recapitulates the exacerbated lung pathology seen after *M. tuberculosis* infection in the complete absence of TNFR1 pathway

**Figure 3:**
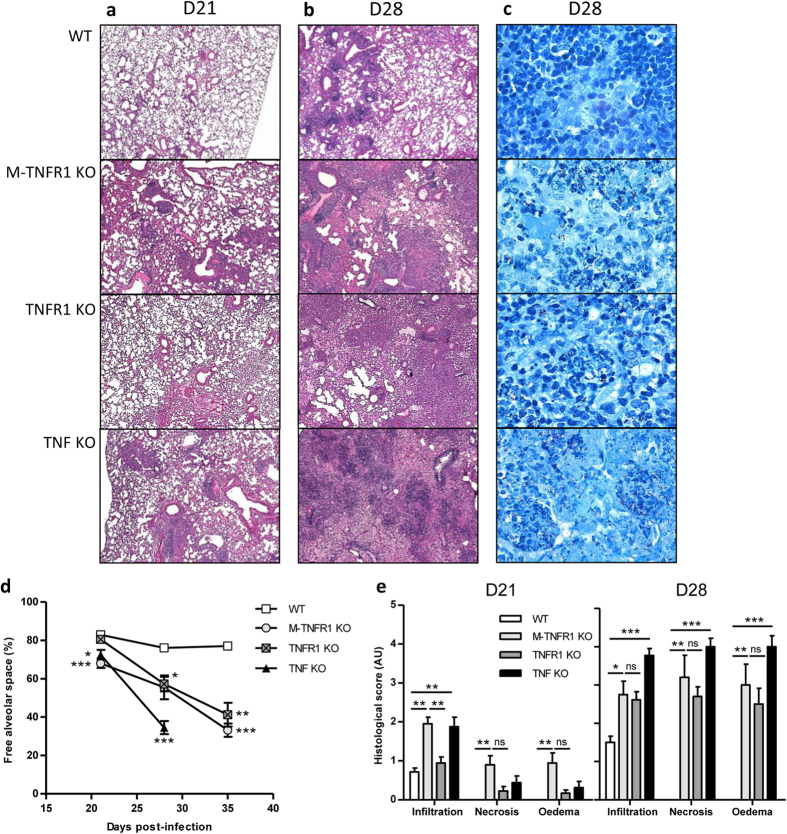
M-TNFR1-deficient mice exhibit acute necrotic pneumonia but defective granuloma formation in response to *M. tuberculosis* infection, similar to TNFR1-deficient mice. Lungs from mice specifically deficient for TNFR1 in myeloid cells (M-TNFR1 KO), TNFR1- or TNFα-deficient mice and wild-type controls were harvested on day 21 and day 28 after *M. tuberculosis* H37Rv infection as in Fig. 2 (1000 CFU/mouse i.n.) and fixed in 4% formol for microscopic analyses (**a–c**). HE staining was performed on day 21 (**a**) and day 28 (**b**). Mycobacteria were visible at day 28 with ZN staining (**c**). HE staining magnification x50, ZN staining magnification x1000. Graphs in (**d**,**e)** summarize free alveolar space and scores of cell infiltration, necrosis and oedema (n = 7–11 from 2 independent experiments); *p < 0.05; **p < 0.01; ***p < 0.001.

Granuloma formation is the result of a structured cell mediated immune response and is associated with efficient containment of *M. tuberculosis* growth. The increased lung inflammation in mice lacking the TNF or TNFR1 pathway prompted us to address the contribution of TNFR1 of myeloid origin in granuloma formation upon *M. tuberculosis* infection. The lungs of TNF deficient mice showed many large confluent nodules at day 28 post infection, which were more discrete in M-TNFR1 KO or in mice fully deficient for TNFR1 and less prominent in wild-type controls. Microscopically, the phenotype of M-TNFR1 KO lungs was visible already at day 21 and developed similarly to that of complete TNFR1 deficient mice, displaying a distinct inflammation with partial reduction of ventilated alveolar spaces, mononuclear cell and neutrophil infiltrations and larger granuloma-type structures than wild-type controls at 28 days post *M. tuberculosis* infection (Fig. 3a,b). TNF deficient mice revealed with large granulomatous inflammatory formations already on day 21, and developed extensive confluent necrosis and oedema by day 28, in the absence of proper granuloma formation. Mycobacteria were visible in the lung of TNFR1 and M-TNFR1 KO mice at that stage (Fig. 3c). They were more pronounced than in wild-type mice but less than those seen within macrophages and in the necrotic area in TNF KO mice, in good agreement with the quantitative assessment of pulmonary bacterial burden (Fig. 2d).

Free alveolar space was already reduced in M-TNF KO and TNF KO 3-weeks post-infection, then similarly in M-TNFR1 KO and TNFR1 KO mice at 4–5 weeks, while WT mice remained stable (Fig. 3d). TNF KO mice were down to 40% free alveolar space at 4 weeks post-infection, when they had to be terminated. Cell infiltration, necrosis and oedema were strongly elevated in M-TNFR1 KO and TNFR1 KO mice at 21–28 days after infection, with a slightly stronger phenotype in M-TNFR1 KO on day 21, similar to TNF KO mice (Fig. 3e). TNF KO mice progressed to further inflammation by day 28, while WT controlled inflammatory cell infiltration, with no necrosis nor oedema.

Thus, the early lung lesions observed in the absence of TNFR1 in myeloid cells were similar to those seen in mice completely deficient for TNFR1 at 4 weeks, suggesting that the contribution of other cells beside myeloid cells to these TNFR1-mediated response is limited at this point, but also that inflammatory myeloid cells do not need to respond to TNF to contribute to lung inflammation.

### Regulation of pulmonary cytokine expression in the absence of myeloid cell TNFR1 pathway after *M. tuberculosis* infection

**Figure 4:**
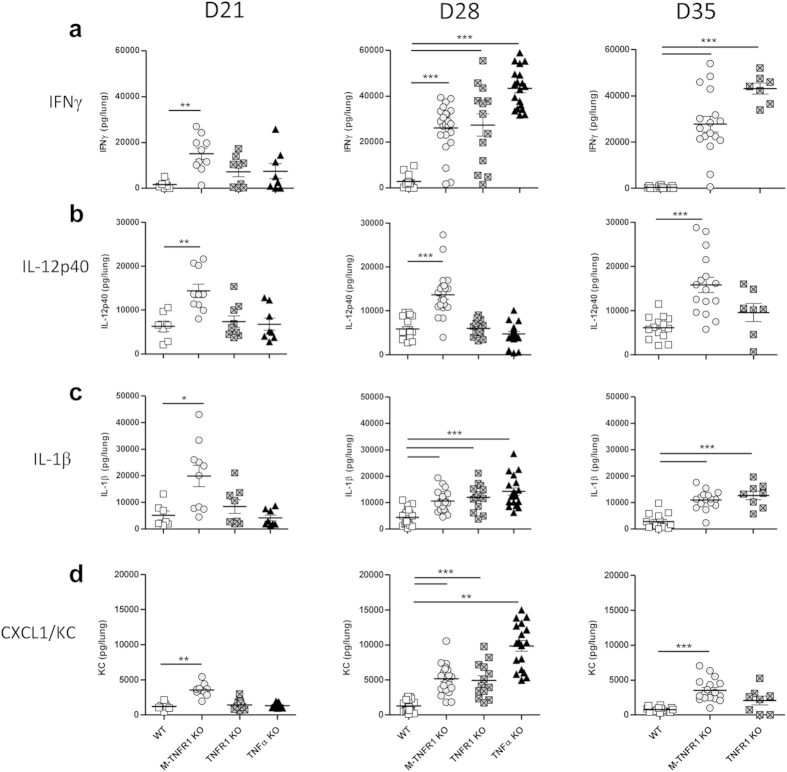
Influence of myeloid TNFR1 expression on cytokine levels in the lungs of *M. tuberculosis* infected mice. Cytokine concentrations were determined in lung homogenates of mice specifically deficient for TNFR1 in myeloid cells (M-TNFR1 KO), TNFR1- or TNFα-deficient mice and wild-type controls 21, 28 or 35 days after *M. tuberculosis* infection, as indicated. IFNγ (**a**), IL-12/IL-23 p40 (**b**), IL-1β (**c**) and CXCL1/KC (**d**) were quantified by ELISA. Results are expressed as single points and mean +/− SEM of n = 7–10 from 2 experiments at day 21, n = 13–24 from 4 experiments at day 28 and n = 8–16 from 3 experiments at day 35. *p < 0.05; **p < 0.01; ***p < 0.001.

As bacilli growth and pulmonary inflammation in myeloid cell-TNFR1 deficient mice was accompanied by increased inflammatory cell infiltration, we next assessed the expression of cytokines and chemokines involved in innate and adaptive immune responses in these mice. Lung IFNγ levels were increased in M-TNFR1 KO mice already at 3 weeks post infection, and further elevated in mice deficient for TNFR1 completely or only on myeloid cells at 4 to 5 weeks after *M. tuberculosis* infection, as well as in TNF deficient mice at 4 weeks when they succumb to the infection (Fig. 4a). IL-12p40 levels were increased in M-TNFR1 KO mice already at 3 and 4 weeks post infection and remained elevated at 5 weeks, a time point when complete TNFR1 KO also exhibited slightly elevated IL-12p40 (Fig. 4b), while they were essentially normal in TNF deficient mice at 3–4 weeks. IL-1α and IL-1β (Fig. 4c) pulmonary levels were elevated in M-TNFRI KO, TNFR1 KO and TNF-deficient mice at 4 weeks of *M. tuberculosis* infection. TNF pulmonary levels were rather modest, as shown previously^26^ and were not affected by the absence of TNFR1. CXCL1/KC levels were increased in M-TNFRI KO already at 3–5 weeks post infection, while this was most prominent at 4 weeks for TNFR1 KO and TNF deficient mice (Fig. 4d). Interestingly, IFNγ, IL-12p40, CXCL1 and IL-1β levels were elevated in M-TNFR1 KO mice already at 3 weeks post infection, indicative of an earlier onset of lung inflammation in these mice.

Therefore, absence of TNFR1 on myeloid cells recapitulates what is seen in complete absence of TNFR1, contributing to the increased pulmonary levels of IFNγ, IL-1β and KC expression during uncontrolled *M. tuberculosis* infection in the absence of TNF. However, myeloid TNFR1 expression seems to contribute to regulate lung IL-12p40 expression after *M. tuberculosis* infection and deletion of TNFR1 in myeloid cells led to earlier inflammation not only histopathologically but also at the molecular level, most notably by higher IL-12p40 pulmonary levels.

### TNFR1-dependent control of granulocytes, the major cellular source of IL-12p40 in the lung after *M. tuberculosis* infection

**Figure 5:**
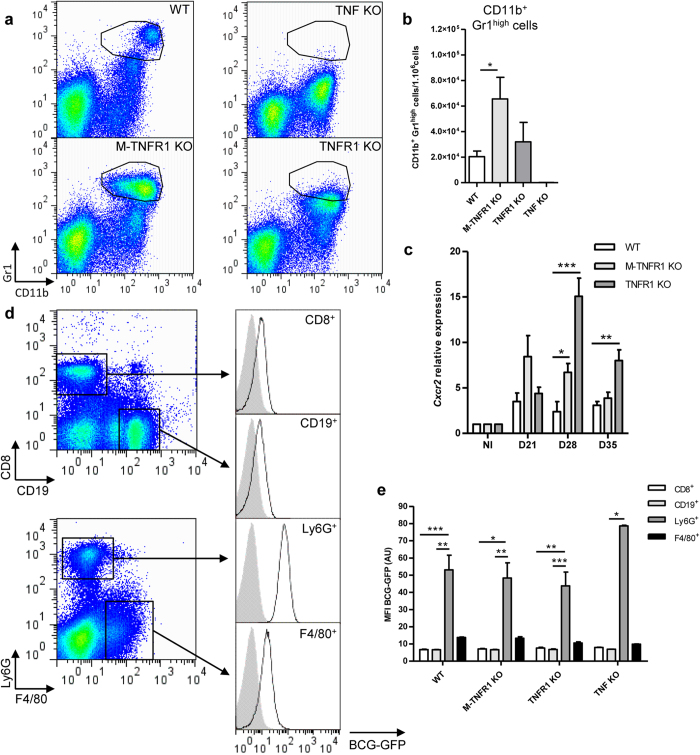
TNFR1 in macrophages/neutrophils regulates recruitment and/or survival of granulocytes, the major mycobacteria internalizing cell population. Lungs from M-TNFR1, fully TNFR1 or TNFα deficient mice and wild-type controls were harvested on day 28 after *M. tuberculosis* H37Rv infection (1000 CFU/mouse i.n.) for flow cytometry analysis. (**a**) Representative dot plots of CD11b^+^/Gr1^+^ cells. (**b**) Bargraph of CD11b^+^ Gr1^high^ cells in *M. tuberculosis*-infected lungs. (**c**) Relative *Cxcr2* gene expression of in the lung of infected M-TNFR1- or TNFR1-deficient mice and wild-type controls. **(d**,**e)** Mice deficient for TNF-, TNFR1 or myeloid TNFR1 and wild type mice were infected with GFP expressing *M. bovis* BCG (2.10^6^ CFU/mouse i.v.) and spleen cells were isolated at day 41 post infection for flow cytometry analysis. (**d**) Representative dot plots of CD8^+^, CD19^+^, Ly6G^+^ and F4/80^+^ cells. Each population was analysed for the presence of BCG-GFP, as compared to isotype control (grey). (**e**) Bar graphs representing mean fluorescence intensity of BCG-GFP in the different spleen cell populations. Results are expressed as mean +/− SEM of n = 4–5 mice per group (**b**,**e**) and n = 4–11 (**c**) from two independent experiments, with n = 2 TNF KO as references. *p < 0.05; **p < 0.01; ***p < 0.001.

We next characterized TNFR1-dependent recruitment of inflammatory cells in the lung after *M. tuberculosis* infection. Lung cells from TNF-, TNFR1-, or M-TNFR1-deficient mice and wild-type controls were analysed by flow cytometry 28 days after infection, at a time when they display inflamed lung (Fig. 2g). M-TNFR1 KO mice showed a higher proportion of pulmonary CD11b^+^ Gr1^high^ cells as compared to TNFR1 KO and WT mice while they were essentially absent in TNF KO mice (Fig. 5a,b). CD11b^+^ Gr1^high^ cells were Ly6C^+^ Ly6G^+^ F4/80^−^, indicating that this population is mostly composed of granulocytes. CD3ε^+^ T cells were unaffected by the absence of TNFR1 (See Supplementary Fig. S2). The increased granulocyte population in the lung of M-TNFR1 KO mice was accompanied by an early over-expression of CXCL1 (Fig. 4f) and of its receptor CXCR2 at day 21 (Fig. 5c), that culminated at 4 weeks in TNFR1 KO mice. Granulocytic CD11b^+^ F4/80^−^Gr1^+^ cells were the major splenic cell population internalizing BCG-GFP after systemic *M. bovis* BCG infection, (Fig. 5d,e).

**Figure 6:**
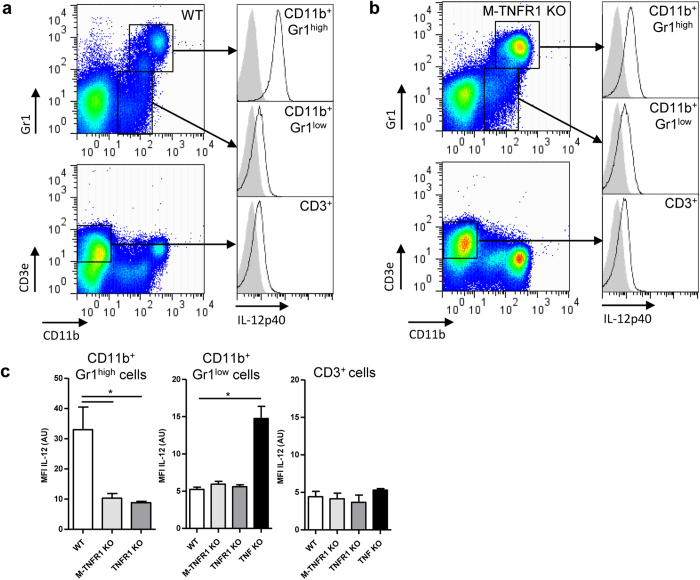
M-TNFR1 dependent IL-12 production in granulocytes from *M. tuberculosis* infected lungs. **(a–c)**. Intracellular IL-12p40 expression in CD11b^+^ Gr1^high^ cells, CD11b^+^ Gr1^low^ cells or in CD3e^+^ cells was represented in WT mice (**a**) and in M-TNFR1 KO mice (**b**). Bar graphs (**c**) summarise IL-12p40 MFI in each cell populations for WT, M-TNFR1-, TNFR1-, or TNFα KO mice. Results are expressed as mean +/− SEM of n = 4–5 mice per group from two independent experiments, with n = 2 TNF KO as references. *p < 0.05; **p < 0.01; ***p < 0.001.

M-TNFR1 KO mice displayed significantly higher levels of IL-12p40 in the lungs after infection (Fig. 4b) and we next characterised IL-12p40 cellular sources. Chimeric, bone marrow reconstituted TNFR1 KO mice revealed that IL-12p40 producing cells are of hematopoietic donor origin (See Supplementary Fig. S3). Further, we show that IL-12p40 expression was highest in CD11b^+^ Gr1^high^ cells as compared to CD11b^+^ Gr1^low^ or CD3ε^+^ *cells (*Fig. 6a,b). However IL-12p40 MFI was reduced in CD11b^+^ Gr1^high^ cells from M-TNFR1 KO and TNFR1 KO mice as compared to WT type mice (Fig. 6c), indicating that lung IL-12p40 overexpression resulted from an increased number of IL-12 producing cells, rather than from a higher IL-12p40 expression per cell. Indeed, primary neutrophils release IL-12p40 in response to inflammatory stimulation *in vitro* and CD11b^+^ F4/80^−^Gr1^+^ cells were also the major cellular source of IL-12p40 in a *M.bovis* BCG model of infection (See Supplementary Fig. S4).

Thus, cell-specific deletion of TNFR1 leads to an increase in pulmonary granulocyte population, the major cellular source of IL-12p40 after mycobacterial infection; IL-12p40 expression being partially dependent on the TNFR1 pathway, the high IL-12p40 levels can be attributed to the increased neutrophil population in the lung of M-TNFR1 KO mice.

### T-cell TNFR1 pathway is dispensable for the control of *M. tuberculosis* infection

**Figure 7:**
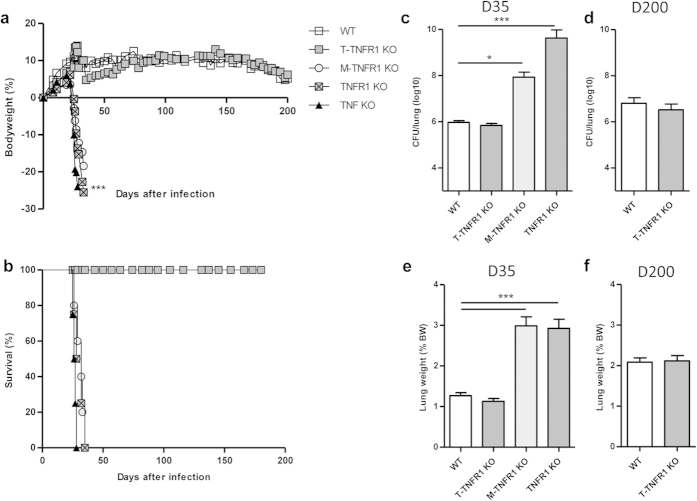
T-TNFR1 KO mice control acute *M. tuberculosis* infection. Mice specifically deficient for TNFR1 in T cells (T-TNFR1 KO) or myeloid cells (M-TNFR1 KO), fully TNFR1 or TNFα deficient mice and wild-type mice were exposed to *M. tuberculosis* H37Rv (1000 CFU/mouse i.n.) and monitored for relative body weight gain (**a**) and survival (**b**). Pulmonary bacterial load (**c,d**) and lung wet weight (**e,f**) were measured at 35 days (**c,e**) and 201 days (**d,f**) post-infection. Results are expressed as mean +/− SEM from n = 9–12 mice per group pooled from 3 independent experiments up to day 35 and n = 9–10 from 2 experiments thereafter. *p < 0.05; **p < 0.01; ***p < 0.001.

TNF from T cell origin is essential for the long-term control of *M. tuberculosis* infection while TNF from myeloid origin is important for the early control of mycobacteria burden^16^, in line with the role of adaptive CD4^+^ T cell response in controlling chronic infection. We next assessed the contribution of TNFR1 pathway in T cells during *M. tuberculosis* infection. Mice deficient for TNFR1 on T cells were obtained by intercrossing Tnfrsf1α conditional knockout mice (*Tnfrsf1*α^*fl/fl*^)^27^ to CD4-Cre transgenic mice, leading to p55 TNFR1 inactivation in all T cells (*Tnfrsf1*α^*fl/fl*^ *CD4*^*cre*^, T-TNFR1 KO). Mice deficient for TNFR1 on T cells survived *M. tuberculosis* infection for up to 200 days, the duration of the experiment, while M-TNFR1 KO and complete TNFR1 KO mice rapidly lost weight and succumbed by day 35 after infection (Fig. 7a,b). Indeed, T-TNFR1 KO mice displayed a similar bacterial load as compared to WT mice 5 weeks post-infection while myeloid cell specific M-TNFR1 deficient and fully TNFR1 KO mice exhibited 2–3log10 increased pulmonary bacterial loads (Fig. 7c). Lung bacterial load in T-TNFR1 KO was similar to that of wild-type mice at 200 days post-infection (Fig. 7d). Absence of TNFR1 on T cells was verified *ex vivo* in the lung of *M.tuberculosis* infected T-TNFR1 KO mice (See Supplementary Fig. S5). Lung inflammation was not augmented in T-TNFR1 KO as compared to wild-type mice, while M-TNFR1 KO and complete TNFRI KO mice exhibited strongly increased lung weights 5 weeks post-infection (Fig. 7e). Lung inflammation in T-TNFR1 KO remained similar to that of wild-type controls at 200 days post infection (Fig. 7f).

**Figure 8:**
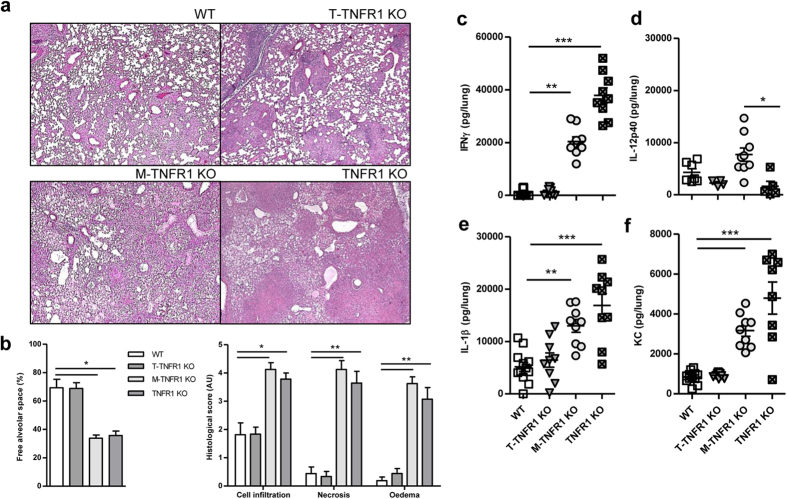
Absence of TNFR1 on T-cells does not increase lung pathology and inflammation after *M. tuberculosis* infection. Lungs from T-TNFR1, M-TNFR1, fully TNFR1 or TNFα deficient mice and wild-type controls were harvested on day 35 post-infection for microscopic analyses and cytokine content. Representative HE staining of lung sections (**a**) (magnification x50). Bar graphs (**b**) summarizing free alveolar space and scores of cell infiltration, necrosis and oedema (n = 9–10 from 2 independent experiments). (**c–f**). Cytokine concentrations were determined by ELISA in the lung homogenates on day 35 for IFNγ (**c**), IL-12/IL-23 p40 (**d**), IL-1β (**e**), and CXCL1/KC (**f**). Results are expressed as single points and mean +/− SEM of n = 9–10 from 2 experiments. *p < 0.05; **p < 0.01; ***p < 0.001.

T-TNFR1 KO and WT mice displayed a controlled cell infiltration and limited tissue damages 35 days after *M.tuberculosis* infection (Fig. 8a) while large necrosis area and granuloma structures were present in M-TNFR1 KO and TNFR1 KO mice, as expected. Indeed, T-TNFR1 KO mice were similar to WT mice with about 70% of free alveolar space, limited cell infiltration and almost no necrosis and oedema (Fig. 8b) while M-TNFR1- and TNFR1 deficient mice exhibited strongly decreased free alveolar space, with higher cell infiltration, necrosis and oedema. We next addressed pulmonary inflammation at the molecular level. IFNγ levels were similar in T-TNFR1 KO and WT mice, although they were highly elevated in the lungs of M-TNFR1 KO mice, 5 weeks after infection (Fig. 8c). Lung IFNγ expression was associated with TCRαβCD4^+^, TCRαβCD8^+^ and CD3^−^NK1.1^+^ cells and was clearly increased in M-TNFR1 KO, TNFR1 KO and TNF KO at day 28, when inflammation is maximal (See Supplementary Fig. S6). The proportions of CD4^+^, CD8^+^ and NK cells were not significantly modified in deficient mice. IFNγ expression was not affected in T-TNFR1 deficient mice, indicating that IFNγ release was independent of TNF/TNR1 response in T cells. Similarly, IL-12p40, IL-1β and KC pulmonary levels were not increased in T-TNFR1 KO mice (Fig. 8d,e,f).

We next asked how the absence of TNFR1 expression on myeloid or T cells would impair the control of a reactivating *M. tuberculosis* infection^28^ (See Supplementary Fig. S7). A Rifampicin plus isoniazid antibiotic treatment given on day 14–35 post infection effectively reduced lung bacterial load by 4 log_10_ in wild-type mice and allowed the survival of susceptible TNF and TNFR1-deficient mice from 4–5 weeks to 11–12 weeks post infection. Six weeks after resuming the antibiotic treatment, TNF KO mice rapidly lost weight, developed severe illness, and were terminated at 11 weeks with lung bacterial loads of 4 log_10_ CFU/lung higher than wild-type mice and severe inflammation indicating *M. tuberculosis* infection reactivation. Similarly, TNFR1 KO mice degraded rapidly and had to be terminated on day 88 post infection with high bacterial burden (5.6 log_10_ CFU/lung) and inflammatory lung. Absence of TNFR1 on myeloid cells yielded a flare of the infection by day 117 in M-TNFR1 mice (6.3 log_10_ CFU/lung) with inflamed lung while T-TNFR1 survived to this point without bodyweight loss, and neither lung inflammation nor bacterial load increase, as compared to wild-type mice.

Therefore, mice deficient for TNFR1 on T cells control acute or reactivating *M. tuberculosis* infection, in contrast to mice deficient for TNFR1 on myeloid cells or complete TNFR1 deficient mice that succumb to primary or flaring infection with highly inflamed lungs, indicating that TNFR1 signalling in T cells is dispensable to mount an effective control of *M. tuberculosis* infection.

## Discussion

TNF is central to control tuberculosis and it is of utmost importance to understand how this occurs^29^,^30^. Indeed, at a time when new drug-resistant TB spreads^1^ developing adjunct host-directed therapies modulating the immune response of the host represents an interesting avenue to fight increasingly drug resistant *M. tuberculosis* infection^31^. Treating severe inflammatory diseases with anti TNF antibodies may compromise host control of TB in patients^10^,^11^,^12^,^13^, as they do in mice^22^. The role of TNF in granuloma formation and maintenance stimulated experimental host directed adjunctive therapies, and TNF neutralization combined with chemotherapy was shown to enhance *M. tuberculosis* bacterial clearance^32^,^33^. Deeper understanding of the multiple facets of TNF mediated TB control may lead to novel approaches of host-directed therapies. Beyond TNF neutralisation, antibody-mediated cell lysis contributes to the mode of action of anti TNF antibodies like Infliximab^34^,^35^. Complete TNF genetic deletion recapitulates the sensitivity to *M. tuberculosis* infection but does not allow to separate the role of TNF in early events of innate immunity versus downstream adaptive responses or contribution from different cell populations. Inducible TNF deletion during established *M. tuberculosis* infection compromised the control of established *M. tuberculosis* infection (VQ, BR, SR unpublished data). Further, huTNF KI mice expressing human TNF in place of murine TNF control *M. tuberculosis* infection^36^, pointing to the role of TNFR1 in this response^22^. The main sources of TNF involved in host control of *M. tuberculosis* are of hematopoietic origin, TNF from myeloid cells being involved in the early control of acute infection while T-cell derived TNF contributes to long-term protection during chronic infection^16^. Since TNFR1 may have autocrine effects, in macrophages or T cells for instance, we addressed the role of TNFR1 from myeloid cells, versus lymphoid cells or parenchyma, non-hematopoietic cells for the host control of *M. tuberculosis* infection. Mice deficient in TNFR1 on myeloid cells recapitulated the dramatic impairment of host response to acute *M. tuberculosis* infection seen in the complete absence of TNFR1, whereas absence of TNFR1 on T-cells or on radio-resistant, parenchymal cells did not affect the outcome of the infection. Thus, using cell-specific deficient mice we demonstrate here for the first time that absence of TNFR1 on macrophages and neutrophils is responsible for the uncontrolled infection seen in complete TNFR1 deficient mice. This suggests a crucial role of innate myeloid cells in terms of both secretion^16^ and response to TNF through TNFR1 in the early steps of infection, while T-cells are less involved in the TNFR1 mediated response to TNF after *M. tuberculosis* infection.

TNFR1 expression is ubiquitous, except for erythrocytes, while TNFR2 expression is restricted to hematopoietic and endothelial cells. Both TNFR1 and TNFR2 are expressed as membrane receptors but also as shed, soluble molecules able to neutralize TNF^37^ or to stabilize it at low concentrations^38^. We showed recently that sTNFR2, through TNF neutralization, down-modulates IL-12p40 expression after *M. tuberculosis* infection, both *in vivo* and *in vitro* in dendritic cells^23^. The expression of both sTNFR1 and sTNFR2 has been documented in serum after *M. bovis* BCG infection^39^ and in the lung of *M. tuberculosis* infected mice^23^. In TNFR1 or TNFR2 deficient mice, beyond the absence of TNF/TNFR mediated signalling pathway, the neutralization of TNF by the corresponding shed, soluble TNFR is also missing. Here, M-TNFR1 KO mice still have a functional TNFR1 pathway in lymphoid, endothelial and epithelial cells with associated release of sTNFR1. Indeed, the levels of pulmonary sTNFR1 were barely affected in M-TNFR1 KO mice and there was no impact of the absence of TNF or TNFR1 on pulmonary sTNFR2 levels (Supplementary Fig. S8), indicating that the bioavailability of TNF should be preserved in these mice.

Interestingly, the pulmonary inflammation manifested earlier and stronger in M-TNFR1 KO mice as compared with complete TNFR1 KO. The levels of IFNγ, IL-12/IL-23 p40, IL-1β, CXCL1/KC as well as the expression of CXCR2 were increased in the lung of M-TNFR1 KO as compared with TNFR1 KO 3 weeks post-infection, and further increased in both groups at 4–5 weeks. This might also be linked to TNFR1 deficiency in other Lys-M expressing cells, beside classical alveolar or recruited macrophages, inflammatory monocytes, monocyte-derived dendritic cells and neutrophils, such as myeloid derived suppressor cells (MDSC), a heterogeneous population with suppressive functions, or alveolar epithelial type 2 cells. Indeed, MDSC have been identified in the lung of mice susceptible to *M. tuberculosis*^40^,^41^. They need TNF for their suppressive function^42^, although this was attributed to TNFR2 pathway^43^ and their recruitment is IL-12-dependent^44^. Here, there was an overexpression of both arginase 1 and nitric oxide synthase transcripts, a hallmark of MDSCs (Knaul *et al.* 2014), in the lung of TNF or TNFR1 deficient mice, M-TNFR1 KO showing an early upregulation of *Nos*2 at 3–4 weeks post-infection, in line with the early IL-12 expression in these mice (Supplementary Fig. S9). Alveolar epithelial type II cell lines may internalize *M. tuberculosis,* undergo autophagy^45^, respond to TNF through TNFR1 or TNFR2^46^,^47^, and express proinflammatory cytokines and chemokines^48^, but the contribution of TNF signal in type II cells for *in vivo* host control to *M. tuberculosis* infection remains unclear.

There was an early and persistent IL-12p40 over-expression in M-TNFR1 KO mice after *M. tuberculosis* infection. Exacerbations of autoimmune diseases under anti TNF therapy was attributed to anti-inflammatory effects of TNF linked to the inhibition of IL-12p40 and IL-23 via TNFR1 on macrophages and dendritic cells^24^. We also report an increased expression of IL-12p40 in CD11b^+^ Gr1^low^ cells from TNF deficient mice, in line with the inhibition of IL-12p40 by TNF in macrophages^49^. Here, we hypothesized that neutrophils were the source of IL-12p40 seen in M-TNFR1 KO. Indeed, IL-12p40 over-expression was associated with an increased population of granulocytic cells, the majority of IL-12 producing cells being F4/80^−^Gr1^+^. Neutrophils are recruited already 1 week post-infection and are most potent to phagocytose mycobacteria, as seen here by BCG-GFP internalisation after systemic infection. Ly6G^+^ granulocytic cells produced more IL-12p40 than F4/80^+^ macrophages, CD8^+^ T cells or B cells, with 5–10-fold higher MFI. IL-12p40 production by Gr1^high^ granulocytic cells was largely dependent on the expression of TNFR1 in these cells, but the increased neutrophil compartment in *M. tuberculosis* infected M-TNFR1 KO mice compensated for the decreased IL-12p40 secretion per cell. The roles of neutrophils in tuberculosis are multifold and potentially conflicting, from mycobacteria killing to bacilli dissemination and adaptive response trigger^50^. The increased inflammation seen in M-TNFR1 KO mice 3 weeks after infection coincides with activation and arrival of T-cells in the lungs^51^. Triggering of TNFR1 pathway can lead to either NFκB activation or cell death such as apoptosis or necroptosis^52^. Thus, in the absence of TNFR1 on myeloid cells, macrophages and neutrophils should not undergo TNFR1-mediated cell death. This, associated with the heavy bacterial burden and high inflammation likely contributed to the increased number of neutrophils seen at day 28 post *M. tuberculosis* infection in M-TNFR1 KO mice. However, it is not excluded that the neutrophil population is regulated indirectly via TNFR1 on macrophages and increased bacterial burden. Uptake of *M. tuberculosis* infected apoptotic neutrophils by dendritic cells has been reported to contribute to their efficient migration to the local lymph nodes and to the activation of Ag-specific CD4 T cells^52^. Conversely, inhibition of neutrophil apoptosis may contribute to the virulence of *M. tuberculosis*, delaying CD4 T cell activation^53^. We therefore propose that TNF, through the TNFR1 pathway in innate myeloid cells, keeps the neutrophil population under control by inducing apoptosis of infected neutrophils that indirectly trigger dendritic cells and adaptive T-cell response. Crosstalks between neutrophils and T cells continue to be unravelled, as shown by the recent report of migrating neutrophils leaving behind fragments of their elongated uropods enriched in CXCL12, a potent T-cell chemoattractant^54^. The fact that neutrophils are the major cellular source of IL-12p40 in the lungs could also contribute to their facilitation of CD4^+^ T cells activation by dendritic cells during *M. tuberculosis* infection^55^. Indeed, the TNF/TNFR1 pathway contributed to trigger IL-12p40 expression by granulocytes. Nevertheless, the higher levels of IL-12p40 were not sufficient to control the infection and were not correlated with higher pulmonary Th1 cell populations as compared to complete TNFR1 KO mice 4 weeks post-infection (See Supplementary Fig. S6). This was despite increased expression of T-cell chemoattractant *Cxcl9* and *Cxcl10* in M-TNFR1 KO mice 3–5 weeks post-infection, likely linked to the increased neutrophil population in these mice (See Supplementary Fig. S9). The expression of arginase 1 was increased in the lungs of *M. tuberculosis* infected TNFR1 KO or M-TNFR1 KO, which may contribute to restrain T cell proliferation^56^. The relative expression of *Tbx21* was significantly reduced in the absence of TNFR1 pathway (See Supplementary Fig. S9), suggesting a defect in differentiation, but not in recruitment, of T-cells in M-TNFR1 KO and TNFR1 KO mice (See Supplementary Fig. S6). Thus, innate cells are critically involved in the response to *M. tuberculosis* in the acute phase, and Th1 cells are not sufficient to control the infection in the absence of myeloid TNFR1 signalling. Indeed, absence of TNFR1 on T-cells did not compromise the control of *M. tuberculosis* infection over 6 months, and did not lead to exacerbation of lung inflammation, cytokine or chemokine release. Further, TNFR1 on myeloid cells was also more crucial than T cell TNFR1 response in a model of reactivation of *M. tuberculosis* infection after control by chemotherapy, where the infection flares in the context of an established adaptive response. This points to a role of T cells independent of TNFR1-mediated response, which may be through TNF production for instance^16^. Thus, absence of TNFR1 on macrophages and neutrophils, and not on T-cells, is responsible for the uncontrolled infection seen in complete TNFR1 deficient mice. A detrimental role of neutrophils in tuberculosis has been proposed^57^ and our data demonstrate the critical role of TNFR1 pathway in keeping the neutrophil population under control, neutrophil apoptosis being central to mount an efficient adaptive T cell response to *M. tuberculosis*.

In conclusion, the protective TNF/TNFR1 pathway essential for controlling acute primary *M. tuberculosis* infection depends on innate myeloid cells such as macrophages and neutrophils, while the TNFR1 pathway in T cells is dispensable. This suggests a crucial role of innate myeloid cells in terms of both secretion and response to TNF in the early steps of infection, while T-cells are less involved in the TNFR1 mediated response to TNF after *M. tuberculosis* infection.

In autoimmune diseases responding to TNF neutralisation, sparing TNF/TNFR1 pathway on myeloid cells, through anti TNF T-cell targeted bi-specific antibodies for instance, should help keeping protection against new exposure to primary, acute TB infection, in patients screened for absence of previous TB exposure. Conversely, adjunct therapies to optimize host defence to TB through host-directed immunomodulation strategies should not be restricted to the adaptive response, and targeting the early myeloid innate immune response might represent an interesting option to boost the first steps of host response to *M. tuberculosis*.

## Materials and Methods

### Mice

Tnfrsf1α conditional knockout mice (*Tnfrsf1*α^*fl/fl*^; see ref 27 for generation and initial characterization) were crossed to *LysM-Cre* or *CD4-Cre* strains to obtain p55 TNFR1 inactivation in myeloid cells (M-TNFR1 KO, *Tnfrsf1*α^*fl/fl*^ *LysM*^*cre/wt*^), or T lymphocytes (T-TNFR1 KO, *Tnfrsf1*α^*fl/fl*^ *CD4*^*cre*^) respectively. All mice, including also mice fully deficient for TNFR1^58^ or TNF^59^ were backcrossed at least 8–10 times on C57BL/6 genetic background and were bred in the Transgenose Institute animal facility (CNRS UPS44, Orleans). C57BL/6 or *Tnfrsf1*α^*fl/fl*^ *LysM*^*wt/wt*^ mice were used as wild-type controls with similar results. For experiments, adult (8–12 week old) animals were kept in isolators in a biohazard animal unit. The infected mice were monitored every day for clinical status and weighed twice weekly. All animal experiments complied with the French Government’s animal experiment regulations and were approved by the “Ethics Committee for Animal Experimentation of CNRS Campus Orleans” (CCO) under number CLE CCO 2012–1001.

### Infection

Aliquots of *M. tuberculosis* H37Rv (Pasteur) kept frozen at −80 °C were thawed, diluted in sterile saline containing 0.05% Tween 20 and clumping was disrupted by 30 repeated aspirations through a 26 gauge needle (Omnican, Braun, Germany). Pulmonary infection with *M. tuberculosis* H37Rv was performed by delivering 1000 ± 200 CFU/lung into the nasal cavities under xylazine-ketamine anaesthesia, and the inoculum size was verified by determining bacterial load in the lungs on day 1 post infection. GFP-expressing *M. bovis* BCG (gift from Dr. V. Snewin, Wellcome Trust London, U.K.) grown to mid-log phase in Middlebrook 7H9 liquid medium (Difco Laboratories, Detroit, Mi.) supplemented with 10% oleic acid/albumin/dextrose/catalase (OADC, Difco Laboratories) and 0.05% Hygromycin (Invivogen) at 37 °C, was stored at −80 °C in 10% glycerol (Sigma), thawed, diluted in sterile saline and injected intravenously at 2.10^6^ CFU/mouse.

### Bone-marrow reconstitution

CD45.2^+^ TNFR1 deficient mice lethally irradiated at a dose of 9 Gy received 3.10^6^ bone-marrow cells (i.v.) from either TNFR1 KO or CD45.1^+^ Ly5.1 WT mice on C57Bl/6 background and their hematopoietic reconstitution followed for 8 weeks before infection with *M. tuberculosis* H37Rv as described above. Conversely, CD45.1^+^ Ly5.1 WT control mice were irradiated and reconstituted with BM cells from either TNFR1 KO or CD45.2^+^ B6 mice and infected as above.

### Bacterial counts

Bacterial loads in tissues were evaluated as described^60^. Organs were weighed and defined aliquots homogenized in PBS in a Dispomix homogenizer (Medic Tools, Axonlab, Baden-Daettwil, Switzerland). Tenfold serial dilutions of organ homogenates in 0.05% Tween 20 containing 0.9% NaCl were plated in duplicates onto Middlebrook 7H11 (Difco) agar plates containing 10% OADC and incubated at 37 °C. Colonies were enumerated at 3 weeks and results are expressed as log_10_ CFU per organ.

### Pulmonary cytokine determination

Lung homogenates were centrifuged (3 min at 14,500 rpm), the supernatants sterilized by centrifugation through 0.22 μm filter (3 min at 14,500 rpm; Costar-Corning, Badhoevedorp, The Netherlands), immediately frozen on dry ice and stored at −80 °C until determination of IL-12/IL-23p40, IL-23p19, IFNγ , IL-10, KC, IL-1α, IL-1β, and TNF levels by ELISA (Duoset R&D Systems, Abingdon, UK).

### mRNA expression

Total RNA was isolated from lung using TRI-Reagent (Sigma). Reverse transcription was performed in with SuperScript^®^III Kit (Invitrogen) and cDNA was subjected to quantitative real-time PCR using primers for *Cxcr2* (Qiagen) and GoTaq^®^ qPCR-Master Mix (Promega). *Actinb* and *Hprt1* expression was used for normalization. Raw data were analyzed using the comparative analysis of relative expression, using ΔΔCt methods^61^.

### Flow cytometry analysis

After perfusion with 0.02% EDTA-PBS, harvested lung tissue was sliced into 1 to 2 mm^3^ pieces and incubated in RPMI 1640 (Gibco, Paisley, Scotland, UK) containing antibiotics (Penicilline 100 U/ml-Streptomycine 100 μg/ml), 10 mM Hepes (Gibco), liberase (62.5 μg/ml), and DNase (50U/ml; Sigma, St Louis, MO). After 45 min of incubation at 37 °C, single-cell suspension was obtained by vigorous pipetting and filtering through 100 μm. Red blood cells were removed using a lysis buffer (BD PharmLyse^®^) before filtering through 20 μm. Cells were washed three times in RPMI 1640 containing 5% SVF and were then stained according to antibody manufacturer protocols. Rat anti-mouse CD11b-FITC (clone M1/70), Gr1-PECy7 (clone RB6-8C5), F4/80-V450 (clone BM8), IL-12-PE (clone C15.6), CD4-APC (clone RMA-5), CD8α-APC-Cy7 (clone 53–6.7) and B220-FITC (clone RA3-6B2), mouse anti-mouse NK1.1-FITC (clone PK136) and hamster anti-mouse CD3ε-PerCP-Cy5.5 (clone 145-2C11), CD11c-APC (clone HL3) and TCRβ-V450 (clone H57-597) were from BD Pharmingen (San Diego, CA). BCG-GFP fluorescence was amplified by a staining with a rabbit IgG anti-GFP A488-conjugated (Life technologies). IFNγ was assessed in lung cells from *M.tuberculosis* infected mice using a mouse IFN-γ Secretion Assay (MACS Miltenyi Biotec). Stained cells were washed twice, fixed with 1% paraformaldehyde (FACS Lysing solution, BD) and analyzed by flow cytometry on a CANTO II analyser (Becton Dickinson). Data were processed with FlowJo software (version 7.6.5 for Windows, FlowJo LLC, Ashland, Oregon).

### Histopathological analysis

Pulmonary left lobe from *M. tuberculosis* infected mice were fixed in 4% phosphate buffered formalin, paraffin-embedded, and 2–3-μm sections stained with haematoxylin and eosin or Ziehl-Neelsen method. The latter involved staining in a prewarmed (60 °C) carbol-fuchsin solution for 10 min followed by destaining in 20% sulphuric acid and 90% ethanol before counterstaining with methylene blue. Free alveolar space, lung cellular infiltration, oedema and necrosis were quantified using a semi-quantitative score with increasing severity of changes (0–5) by two independent observers including a trained pathologist (BR).

### Statistical analysis

Statistical significance was determined with Graph Pad Prism (version 5.04 for Windows, GraphPad Software, La Jolla, CA). Differences between multiple *in vivo* groups were analysed by means of one-way non-parametric ANOVA test (Kruskal-Wallis followed by Dunn’s multiple comparison test, alpha level = 0.05) and values of *p* ≤ 0.05 were considered significant. Two-tailed, non-parametric Mann-Whitney test was used for analysing *in vitro* results.

## Additional Information

**How to cite this article**: Segueni, N. *et al.* Innate myeloid cell TNFR1 mediates first line defence against primary *Mycobacterium tuberculosis* infection. *Sci. Rep.* **6**, 22454; doi: 10.1038/srep22454 (2016).

## Supplementary Material

Supplementary Information
