# Supplementary material for: Innate myeloid cell TNFR1 mediates first line defence against primary Mycobacterium tuberculosis infection
Source: Sci Rep. 2016 Mar 2;6:22454. doi: 10.1038/srep22454 (PMC4773807; doi:10.1038/srep22454)
Supplement: Supplementary Information [file srep22454-s1.pdf]

Innate myeloid cell TNFR1 mediates first line defence against primary *Mycobacterium tuberculosis* infection.

Noria Segueni[1,2], Sulayman Benmerzoug[1,2], Stéphanie Rose[1,2], Amandine Gauthier[2], Marie-Laure Bourigault[1,2], Flora Reverchon[1,2], Amandine Philippeau[1,2], François Erard[1,2], Marc Le Bert[1,2], Hélène Bouscayrol[3], Thierry Wachter[3], Irène Garcia[4], George Kollias[5], Muazzam Jacobs[6,7], Bernhard Ryffel[1] and Valerie F.J. Quesniaux[1]*

[1] CNRS, UMR7355, Experimental and Molecular Immunology and Neurogenetics, 45071 Orleans, France

[2] University of Orleans, 45000 Orleans, France

[3] Oncology–radiotherapy Department, Regional Hospital, Orleans, France

[4] Department of Pathology and Immunology, University of Geneva Medical School, Geneva, Switzerland

[5] Institute for Immunology, Biomedical Sciences Research Center "Alexander Fleming", Vari, Greece

[6] Division of Immunology, Department of Pathology and Institute of Infectious Disease and Molecular Medicine, Health Sciences Faculty, University of Cape Town, South Africa

[7] National Health Laboratory Service, South Africa

Running title: TNFR1 from myeloid cells in mycobacterial infections

*Corresponding author:

Valerie Quesniaux INEM UMR7355, 3B rue de la Ferollerie, 45071 Orleans, France

Tel : +33 2 38 25 54 39   Fax : +33 2 38 25 79 79

e-mail: quesniaux@cnrs-orleans.fr

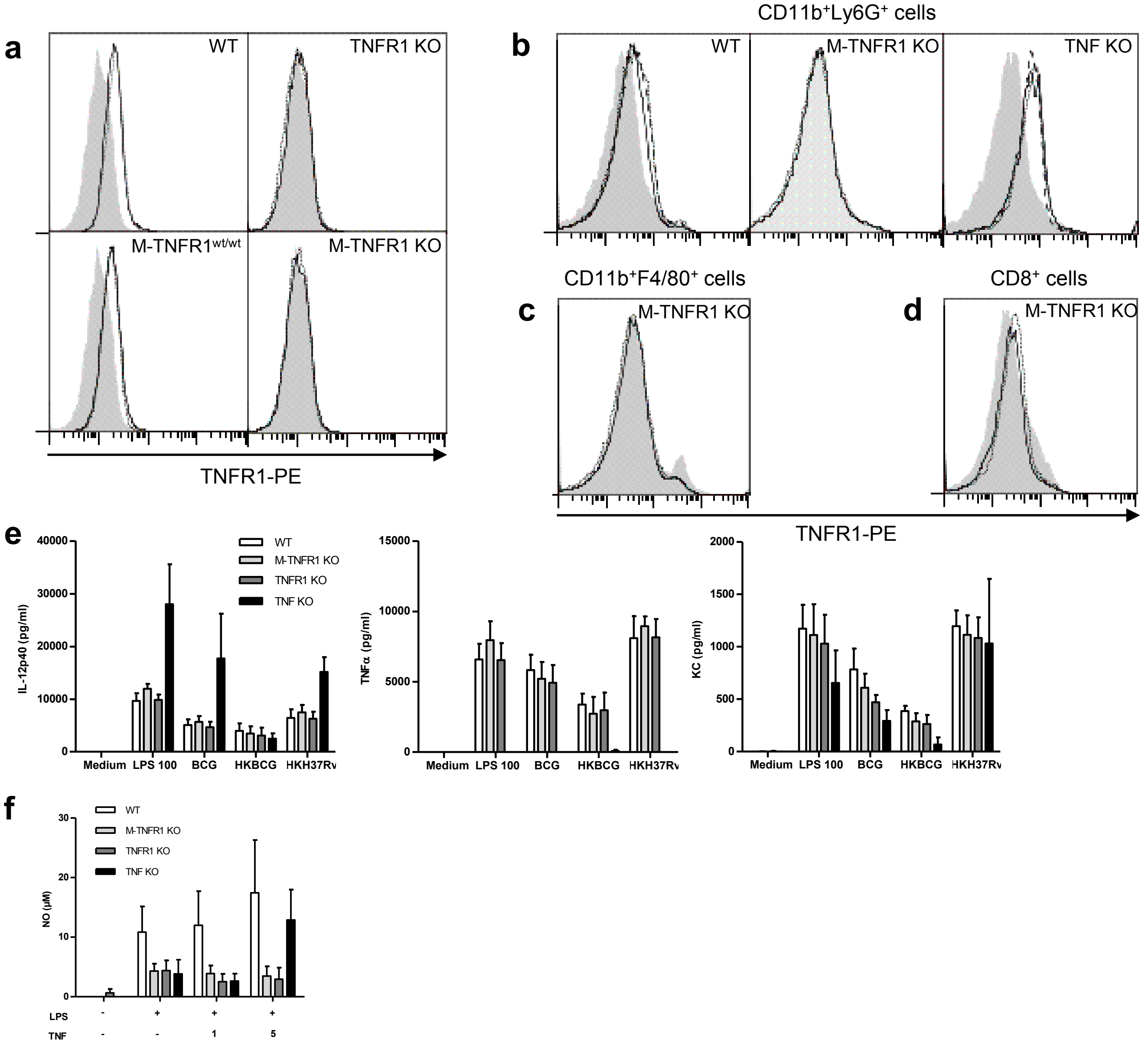

Figure S1. Macrophages from M-TNFR1 KO mice do not express functional TNFR1 but respond to mycobacterial stimuli.

(a) Bone marrow derived macrophages (BMDM) from C57BL/6 (WT), TNFR1 KO, *Tnfrsf1α$^{fl/fl}$ LysM$^{wt/wt}$* (M-TNFR1 $^{wt/wt}$) or *Tnfrsf1α$^{fl/fl}$ LysM$^{cre/wt}$* (M-TNFR1 KO) mice were incubated with hamster α-mouse TNFR1/CD120a and revealed with biotinylated α-hamster IgG plus PE-streptavidine (n=2, dark line and dotted line), as compared to isotype controls (grey). (**b-d).** Spleen cells from WT, M-TNFR1 KO or TNF KO mice were isolated and stained for cytometry analysis 41 days after *M. bovis* BCG infection. TNFR1/CD120a expression was measured in CD11b$^+$Ly6G$^+$ cells (**b**), CD11b$^+$F4/80$^+$ cells (**c**) and CD8$^+$ cells (**d**). (**e)** BMDM from WT, M-TNFR1-, TNFR1- or TNF KO mice were stimulated with LPS (100ng/ml), or live or heat-killed mycobacteria (MOI 2) for 20h. IL-12p40, TNF and KC were measured in supernatants. Results are mean +/- SEM of n=6 mice per group, from 3 independent experiments (**f)** BMDMs were primed with TNF at 5ng/ml for 16h prior to LPS stimulation (at 1ng/ml) for 18h and NO measured in supernatants. Results are mean +/- SEM of n=4 mice per group from 2 independent experiments.

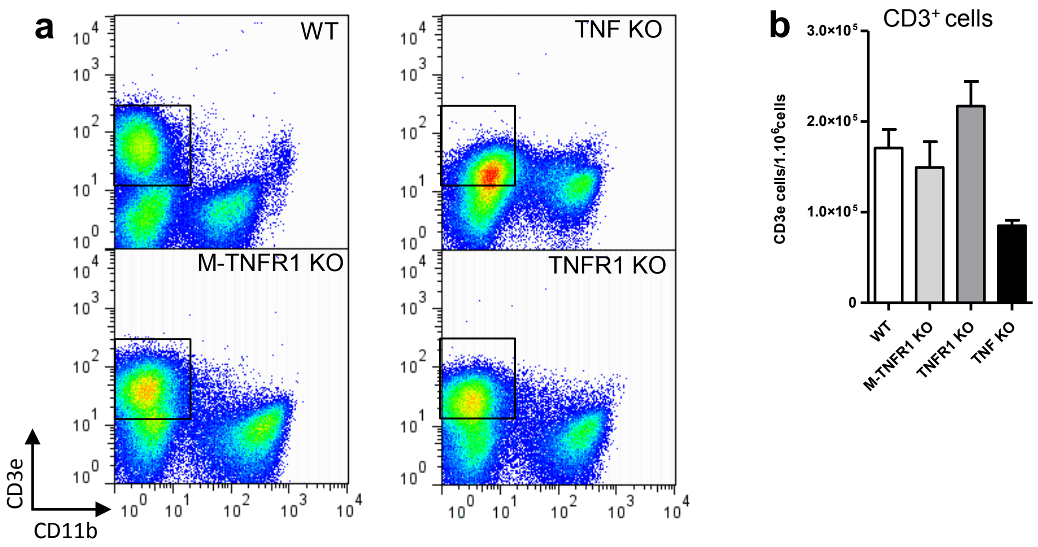

Figure S2. Pulmonary T-cells recruitment after *M. tuberculosis* infection independant of TNFR1 signalisation

Lungs from M-TNFR1-, TNFR1- or TNFα-deficient mice and wild-type controls were harvested on day 28 after *M. tuberculosis* H37Rv infection (1000 CFU/mouse i.n.) for flow cytometry analysis. (**a**) Representative dot plots of $CD11b^+CD3\varepsilon^+$ cells. (**b**) Bargraph of $CD3\varepsilon^+$ cells in the lungs of M-TNFR1-, TNFR1-, or TNFα-deficient mice and wild-type controls. Results are expressed as mean +/- SEM of n=2-5 mice per group from two independent experiments. *, p<0.05; **, p<0.01; ***, p<0.001.

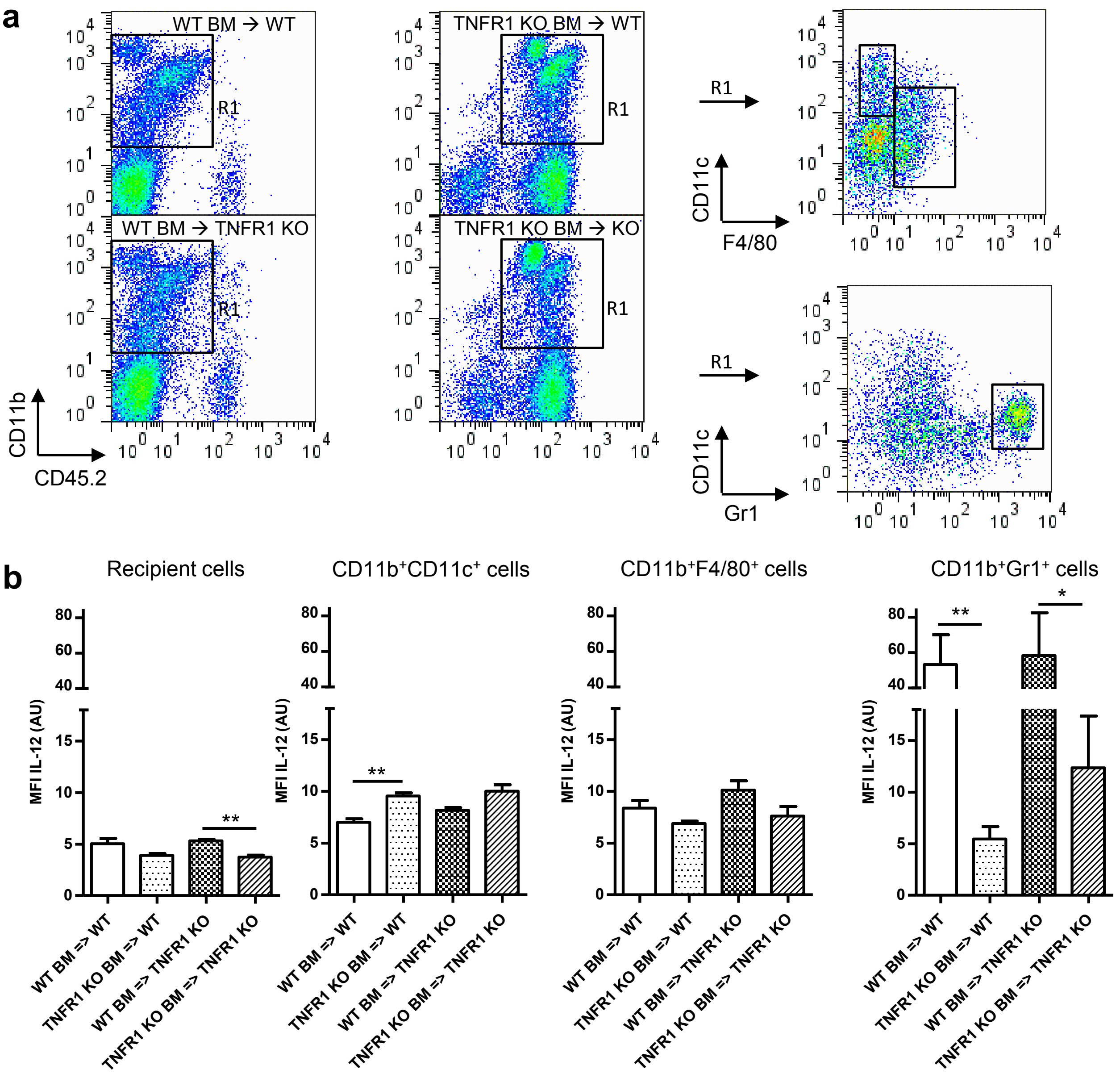

Figure S3. Hematopoietic cells are the major source of IL-12p40 in the lungs after *M. tuberculosis* infection.

TNFR1 deficient mice and WT mice were lethally irradiated and reconstituted with bone marrow from either WT mice or TNFR1 KO mice before *M.tuberculosis* intranasal infection. Lungs were harvested 30 days post-infection and stained for CD45.2, CD11b, CD11c, F4/80, Gr1 and IL-12p40. (**a**) Gating strategy used for IL-12p40 MFI determination is shown as CD45.2/CD11b, F4/80/CD11c and Gr1/CD11c dot plots. (**b**) Bargraphs of IL-12p40 mean fluorescence intensity in each cell population, including cells of recipient origin, and donor $CD11b^+CD11c^+$, $CD11b^+F4/80^+$ and $CD11b^+Gr1^+$ cells. Results are expressed as mean +/- SEM (n=6-7 mice per group). *, p<0.05; **, p<0.01; ***, p<0.001.

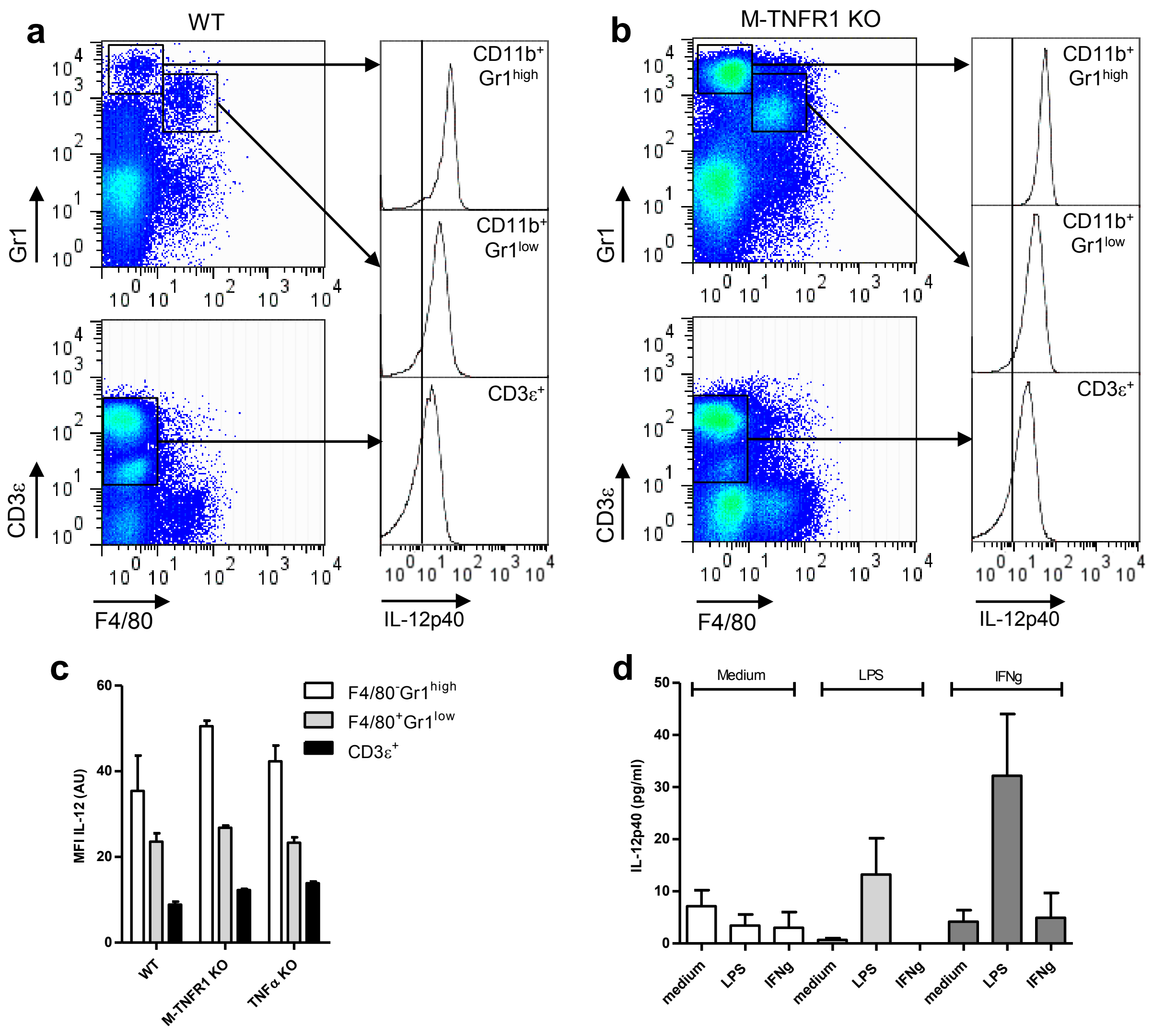

Figure S4. Splenic neutrophils express IL-12p40 after *M.bovis* BCG infection

(a-c) Intracytoplasmic IL-12p40 staining was measured in splenic CD11b^{+}Gr1^{high}, CD11b^{+}Gr1^{low} or in CD3e^{+} cells from WT (**a**) and M-TNFR1 KO mice (**b**). IL-12p40 mean fluorescence intensity (MFI) is represented as bar graph in WT, M-TNFR1 KO and TNF KO mice (**c**). (**d**) Neutrophils isolated from C57BL/6 mice bone marrow were primed with LPS or IFNg for 12 hours prior to stimulation with LPS or IFNg for 12 hours. Supernatants were harvested for IL-12p40 determination by ELISA. Results are expressed as mean +/- SEM of n=3-6 pooled from 2 independent experiments. *, $p<0.05$; **, $p<0.01$; ***, $p<0.001$.

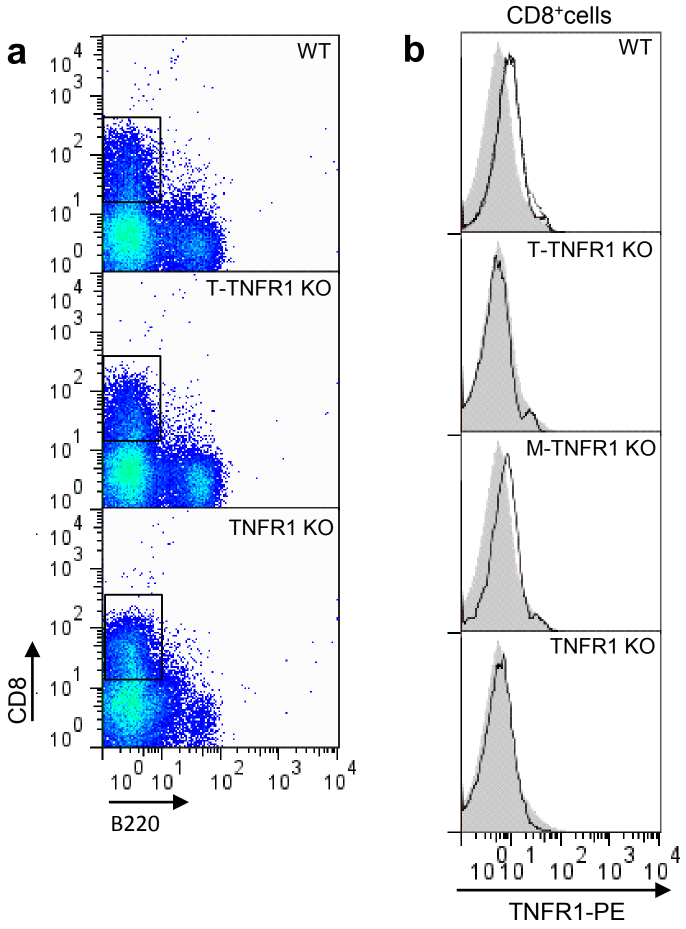

Figure S5. Lymphocytes from T-TNFR1 KO mice do not express TNFR1 ex-vivo

Lung cells from WT and T-TNFR1-, M-TNFR1-, and TNFR1-deficient mice infected with *M. tuberculosis* H37Rv (1000 CFU/mouse i.n.) were isolated on day 28 and stained for CD8 and B220. (**a**) Representative dot plots for CD8^{+} B220^{+} cells are shown for each experimental group. (**b**) TNFR1 expression was measured as mean fluorescence intensity in CD8+ cells as compared to isotype control (in grey).

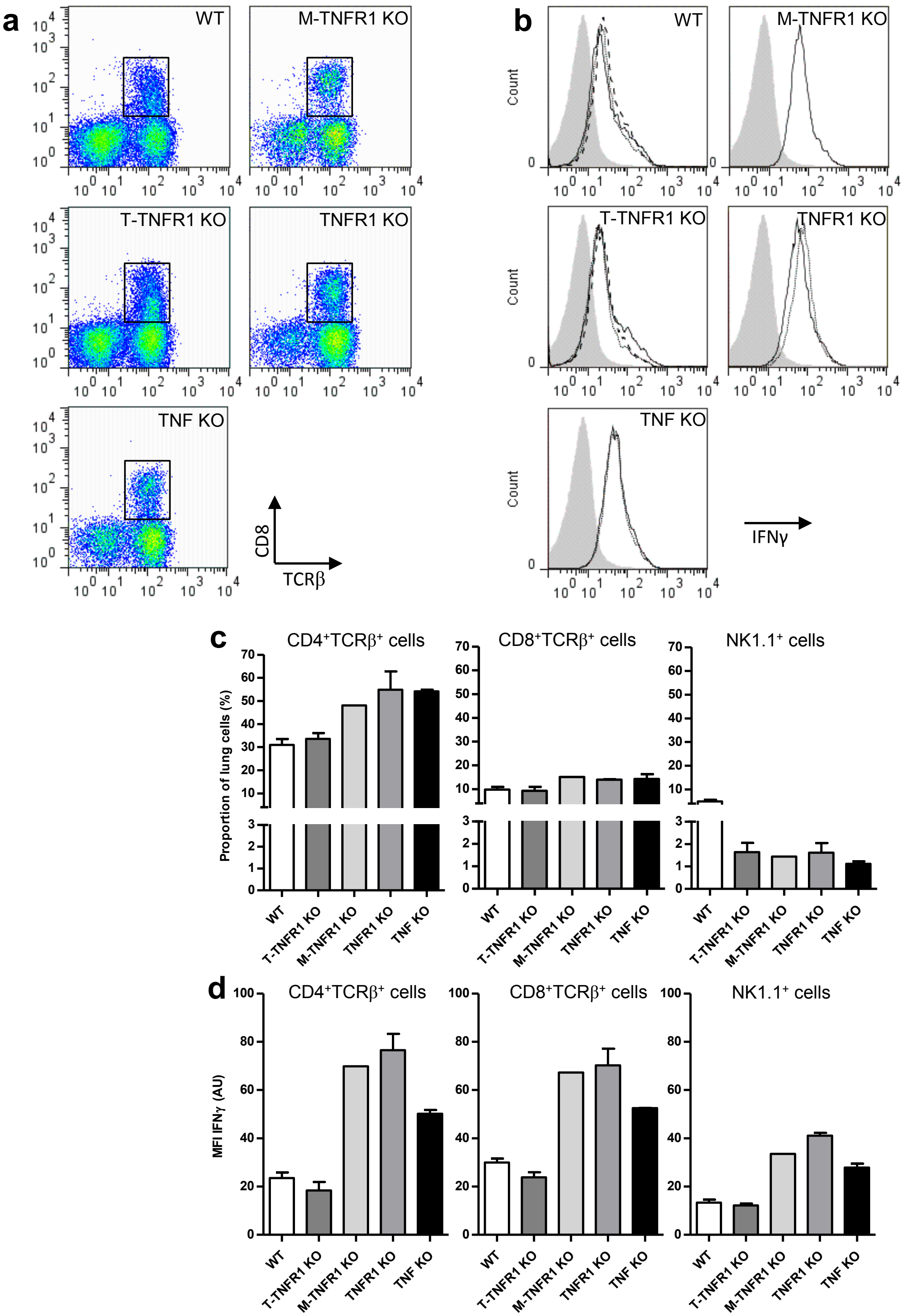

Figure S6. High IFNγ secretion is correlated with susceptibility to *M.tuberculosis* infection but does not require TNF/TNFR1 pathway.

Lung cells from WT and TNF-, TNFR1-, M-TNFR1-deficient mice infected with *M. tuberculosis* H37Rv (1000 CFU/mouse i.n.) were harvested on day 28 and stained for CD3ε, CD4, CD8, TCRβ, NK1.1 and IFNγ using a secretion kit. (**a**) Representative dot plots for CD3ε$^+$ TCRαβCD8$^+$ cells are shown for each experimental group. (**b**) Secreted IFNγ intensity in TCRαβCD8$^+$ cells was plotted for each group (n=2-3, dark, dotted and dashed lines) as compared to isotype control (in grey). (**c-d**) The percentages of CD3ε$^+$ CD4$^+$TCRαβ, CD3ε$^+$ CD8$^+$TCRαβ cells and CD3ε$^-$NK1.1$^+$ cell populations (**c**) and IFNγ MFI (**d**) are represented as bar graphs. Results are expressed as mean +/-SEM of n=2-3 mice.

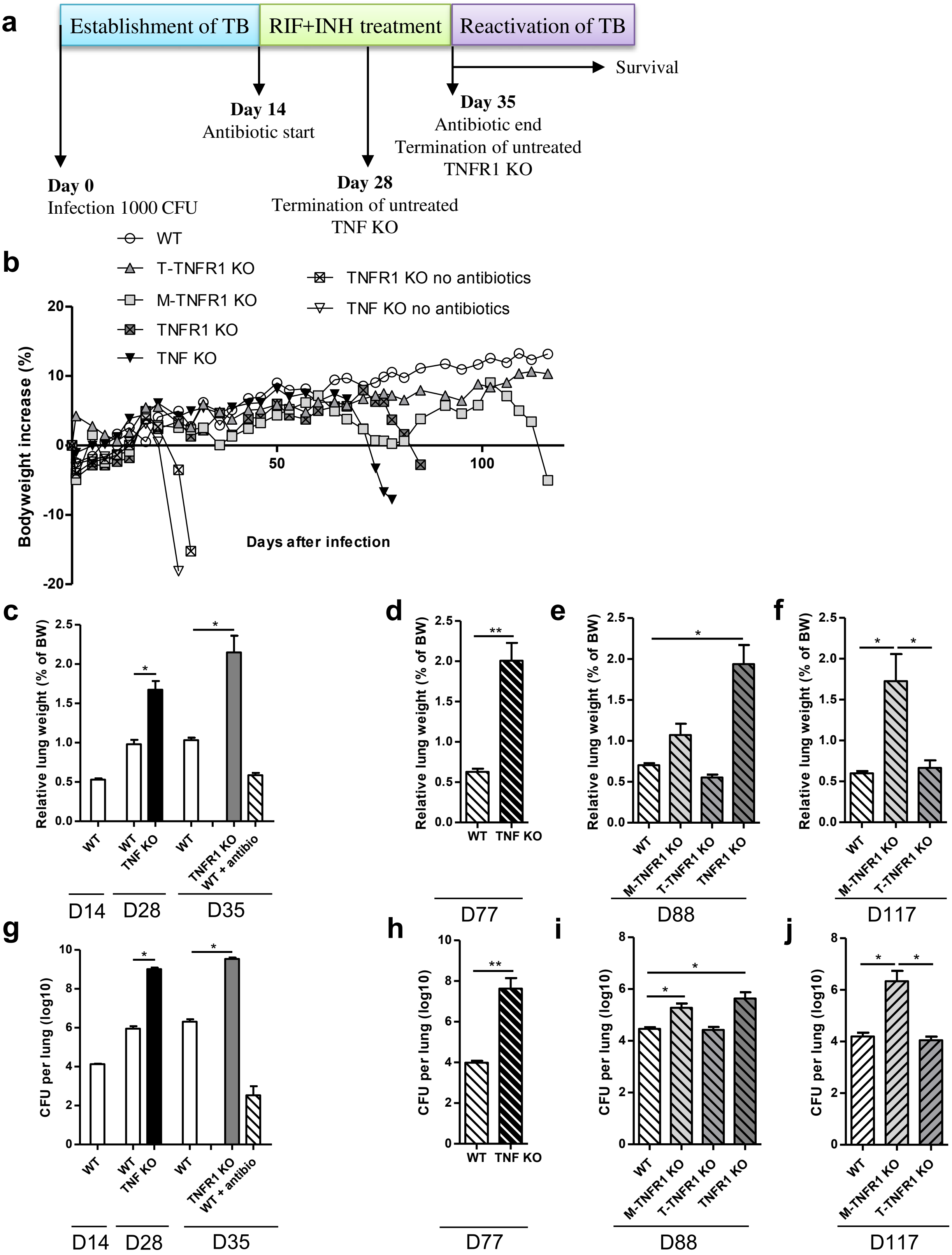

Figure S7. Lack of TNFR1 signalling in myeloid cells leads to a reactivation of *M.tuberculosis* infection
M-TNFR1-, T-TNFR1-, TNFR1 and TNF-deficient mice and wild-type controls were exposed to *M. tuberculosis* H37Rv and treated with isoniazid (INH) and rifampicin (RIF) antibiotics (antibio; each 0.1g/L in drinking water) from 14 days to 35 days post-infection (**a**). Mice were then monitored for bodyweight (**b**). Relative lung weight (**c**, **d**, **e**, **f**) and bacterial load (**g**, **h**, **i**, **j**) were assessed at the indicated days. Results are expressed as mean +/- SEM from n=4-6 mice per group. *, $p<0.05$; **, $p<0.01$; ***, $p<0.001$.

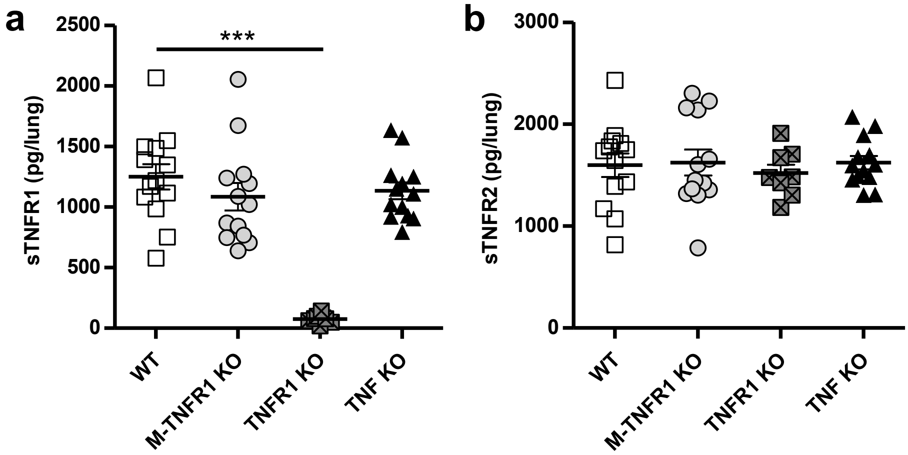

Figure S8. Soluble TNFR1 and TNFR2 levels in the absence of TNFR1 on myeloid cells.

Soluble TNFR1 (**a**) and soluble TNFR2 (**b**) concentrations were determined in lung homogenates of mice deficient for TNF, TNFR1, or myeloid-TNFR1 and wild-type mice 28 days after *M. tuberculosis* infection. Results are expressed as single points and mean +/- SEM of n=8-13 from 3 experiments. *, $p<0.05$; **, $p<0.01$; ***, $p<0.001$.

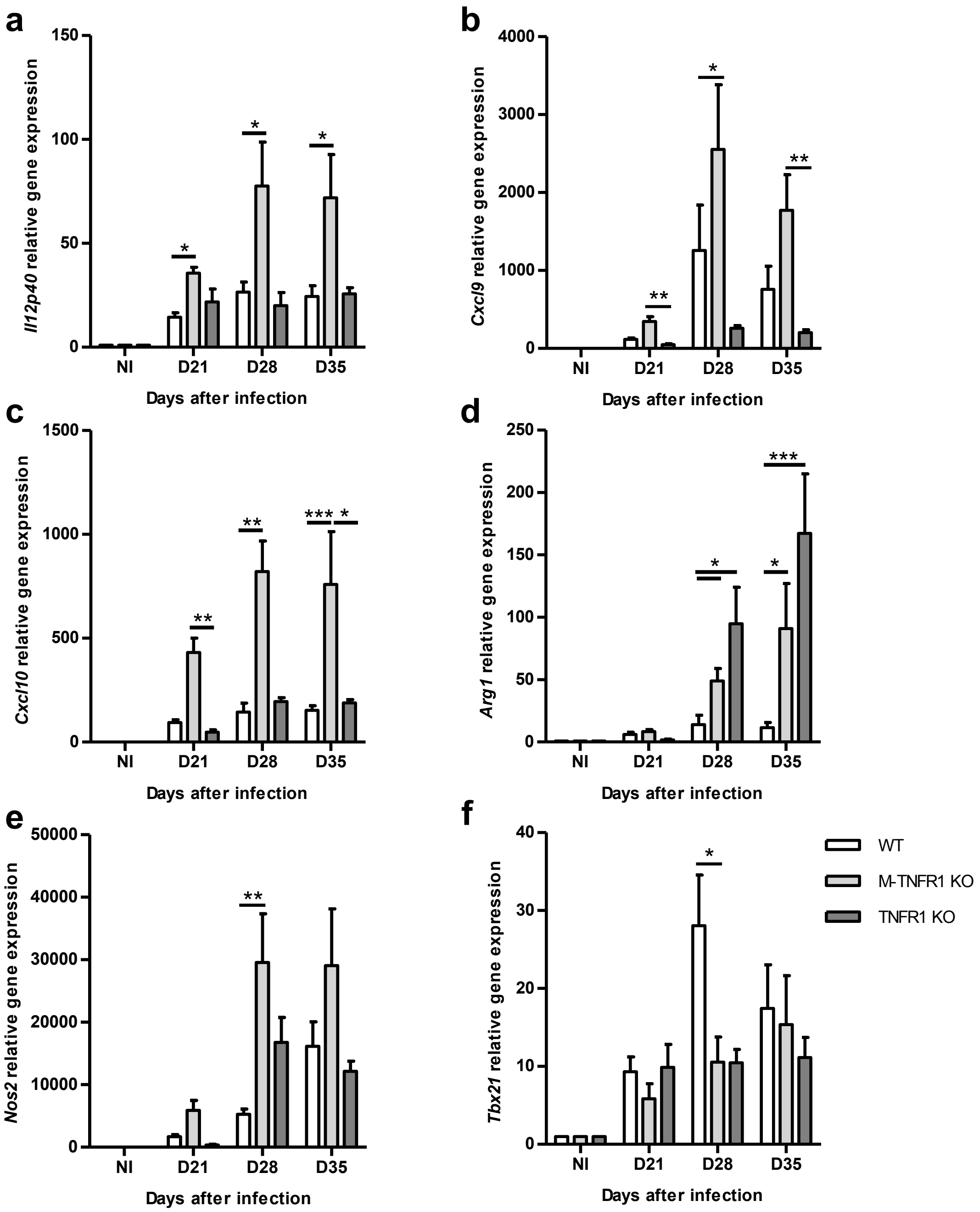

Figure S9. T-cell adaptive response-related gene expression in the absence of TNFR1 pathway. Relative gene expression of *Il12p40* (**a**), *Cxcl9* (**b**), *Cxcl10* (**c**), *Arg1* (**d**) *Nos2* (**e**) and *Tbx21* (**f**) were determined in the lung of TNF, TNFR1, or myeloid-TNFR1 deficient mice and wild-type mice 21, 28 and 35 days after *M. tuberculosis* infection. Results are expressed as mean +/- SEM of n=4-12 from 2 independent experiments. *, $p<0.05$; **, $p<0.01$; ***, $p<0.001$.

Supporting information

Macrophage cultures.

Murine bone marrow cells isolated from femurs were differentiated into macrophages after culturing at 10^6 cells/ml for 7 days in DMEM (Sigma) supplemented with 10 mM L-glutamine, 25mM Hepes, 100U/mL Penicillin and 100U/ml Streptomycin, plus 20% horse serum and 30% L929 cell-conditioned medium as a source of M-CSF [1]. Three days after washing and re-culturing in fresh medium, the cell preparation contained a homogenous population of macrophages. Macrophages were plated in 96 well microculture plates (at 10^5 cells/well in supplemented DMEM as above), and stimulated with LPS (*Escherichia coli*, serotype O111:B4, Sigma, St Louis, MO, at 100 ng/ml), heat-killed *M. tuberculosis* H37Rv (heat-killed 90 min at 80°C), live or heat-killed *M. bovis* BCG (HKBCG; from Pasteur Institute, Paris), at a MOI of 2 bacteria per cell. Cell supernatants were harvested after 24 h of stimulation for IL-12p40, CXCL1/KC and TNF quantification by ELISA (R&D Duoset). For assessing functional TNFR1 response, cells were plated as described and incubated with TNF for 16h before LPS/IFNγ stimulation for 20h [2]. Supernatants were harvested for nitric oxide determination by Griess reaction as described [3].

Reactivation model.

In a pharmacological model of *M. tuberculosis* infection reactivation adapted from the original Cornell model [4], mice were infected with *M. tuberculosis* H37Rv (1000 CFU i.n.) for 2 weeks, and treated with isoniazid (INH) and rifampicin (RIF) (each 0.1g/L in drinking water) for 3 weeks to control the infection, as described[4] (Figure S7). Under this protocol, the bacterial burden is drastically reduced, and the infection may reactivate thereafter in the presence of a primed anti *M. tuberculosis* immune response.

References

1 Muller, M. *et al.* Correction or transfer of immunodeficiency due to TNF-LT alpha deletion by bone marrow transplantation. *Mol Med* **2**, 247-255. (1996).

2 Zakharova, M. & Ziegler, H. K. Paradoxical anti-inflammatory actions of TNF-alpha: inhibition of IL-12 and IL-23 via TNF receptor 1 in macrophages and dendritic cells. *J Immunol* **175**, 5024-5033, doi:175/8/5024 [pii] (2005).

3 Green, L. C. *et al.* Analysis of nitrate, nitrite, and [15N]nitrate in biological fluids. *Anal Biochem* **126**, 131-138 (1982).

4 Botha, T. & Ryffel, B. Reactivation of Latent Tuberculosis Infection in TNF-Deficient Mice. *J Immunol* **171**, 3110-3118 (2003).
